# Nonalcoholic fatty liver disease is associated with the development of obstructive sleep apnea

**DOI:** 10.1038/s41598-021-92703-0

**Published:** 2021-06-29

**Authors:** Goh Eun Chung, Eun Ju Cho, Jeong-Ju Yoo, Young Chang, Yuri Cho, Sang-Hyun Park, Dong Wook Shin, Kyungdo Han, Su Jong Yu

**Affiliations:** 1 Department of Internal Medicine and Healthcare Research Institute, Seoul National University Hospital Healthcare System Gangnam Center, Seoul, Republic of Korea; 2 Department of Internal Medicine and Liver Research Institute, Seoul National University College of Medicine, 101 Daehak-no, Jongno-gu, Seoul, 03080 Republic of Korea; 3 Department of Gastroenterology and Hepatology, Soonchunhyang University Bucheon Hospital, Bucheon, Gyeonggi-do Republic of Korea; 4 Department of Gastroenterology and Hepatology, Soonchunhyang University Seoul Hospital, Seoul, Republic of Korea; 5 Center for Liver and Pancreatobiliary Cancer, National Cancer Center, Goyang, Republic of Korea; 6 Department of Biostatistics, College of Medicine, Soongsil University, Seoul, Republic of Korea; 7 Department of Family Medicine/Supportive Care Center, Samsung Medical Center Supportive Care Center, Samsung Comprehensive Cancer Center, Seoul, Republic of Korea; 8 Center for Clinical Epidemiology, SAIHST, Sungkyunkwan University, Seoul, Republic of Korea

**Keywords:** Non-alcoholic fatty liver disease, Obesity

## Abstract

Increasing evidence suggests that obstructive sleep apnea (OSA) is a metabolic syndrome-related disease; however, the association between nonalcoholic fatty liver disease (NAFLD) and OSA is not firmly established. In this study, we investigated the relationship between NAFLD and OSA in a general population drawn from a nationwide population-based cohort. Data from the Korean National Health Insurance System between January 2009 and December 2009 were analyzed using Cox proportional hazards model. NAFLD was defined as a fatty liver index (FLI) ≥ 60 in patients without excessive alcohol consumption (who were excluded from the study). Newly diagnosed OSA during follow-up was identified using claims data. Among the 8,116,524 participants, 22.6% had an FLI score of 30–60 and 11.5% had an FLI ≥ 60. During median follow-up of 6.3 years, 45,143 cases of incident OSA occurred. In multivariable analysis, the risk of OSA was significantly higher in the higher FLI groups (adjusted hazard ratio [aHR] 1.15, 95% confidence interval [CI] 1.12–1.18 for FLI 30–60 and aHR 1.21, 95% CI 1.17–1.26 for FLI ≥ 60). These findings were consistent regardless of body mass index and presence of abdominal obesity. In conclusion, a high FLI score may help identify individuals with a high risk of OSA. Understanding the association between NAFLD and OSA may have clinical implications for risk-stratification of individuals with NAFLD.

## Introduction

Obstructive sleep apnea (OSA) is characterized by recurrent collapse of the upper respiratory tract during sleep, leading to intermittent hypoxia, snoring, and sleep fragmentation. It is a prevalent disease, affecting 7–20% of the general population^1^ and up to 48–70% of obese populations^2,3^. Cumulative studies have shown that OSA is associated with various metabolic diseases, including hypertension^4^, glucose intolerance^5^, and dyslipidemia^6^, as well as increased all-cause mortality^7^.

Nonalcoholic fatty liver disease (NAFLD) is the most common cause of chronic liver disease worldwide. Its prevalence is increasing and is now as high as 20–30% in Asia^8^. NAFLD may progress in some instances to steatohepatitis, hepatic fibrosis, and cirrhosis^9^ and hepatic steatosis is frequently associated with other disorders, including cardiovascular disease^10^, diabetes^11^, chronic kidney disease^12^, and colorectal cancer^13^. Like OSA, NAFLD is closely related to obesity^14^. In particular, the hallmark of OSA—chronic intermittent hypoxia during sleep—has been associated with NAFLD^15–17^.

Previous studies regarding the association between NAFLD and OSA were conducted to investigate the risk of hepatic steatosis in patients with OSA. The risk of OSA in patients with NAFLD, however, has received little attention. Since the prevalence of diagnosed OSA in patients with NAFLD is relatively low in Asian countries^18^, it is difficult to evaluate the risk of OSA in patients diagnosed with NAFLD. Thus, we used a nationwide population-based cohort of participants in a health screening program to investigate the risk of incident OSA during follow-up in people with hepatic steatosis.

## Methods

### Data source

In this study, we obtained information from the database of the Korean National Health Insurance System (NHIS), which is the national insurer managed by the Korean government and to which approximately 97% of the Korean population subscribes^19^. The NHIS database contains health records, including sociodemographic data (age, sex, and income level), medical diagnosis (based on International Classification of Diseases, 10th revision [ICD-10]), treatment data, and health examination results (lifestyle and laboratory test results), for the Korean population. NHIS recommends that subscribers undergo a standardized medical examination at least biennially. Eligible members can get the examination at medical institutions including private clinics and hospitals, and public health centers engaged voluntarily for the national screening program.

### Study sample

A total of 10,505,818 individuals underwent an annual or biennial evaluation provided by the NHIS in 2009. Patients who met the following criteria were excluded from the study: < 20 years of age (n = 15,317); excessive alcohol consumption (≥ 30 g of alcohol/day) (n = 679,743); diagnosis of liver cirrhosis (K703, K746) or any hepatitis (B15-B19) (n = 989,161), cancer (n = 118,243), or OSA (G473) (n = 25,771); or incomplete information (n = 561,059); these data included smoking history (n = 63,282), exercise (n = 158,986), alcohol history (n = 160,159), body mass index (n = 12,966), waist circumference (n = 7,576), blood pressure (n = 12,752) and laboratory findings (n = 145,338).

The study protocol was approved by the Institutional Review Board of Seoul National University Hospital (E-2002-015-1098) and conformed to the ethical guidelines of the World Medical Association Declaration of Helsinki. The requirement for informed consent from individuals was waived because de-identified secondary data were used.

### Clinical parameters and biochemical analysis

As described previously^20^, standardized self-reported questionnaires and, clinical parameters including laboratory tests were used to collect data at the time of enrollment. Briefly, age, sex, smoking status (non-smoker, ex-smoker, and current smoker), and alcohol consumption (frequency [0–7 days/week] and amount consumed on one occasion) were used. Regular physical exercise was defined as engaging in exercise on a routine basis with moderate to high-intensity activity ≥ 3 times/week. Income level was dichotomized at the lowest 20%. Comorbidities were defined using ICD 10 diagnosis codes, prescription information in the year prior to health screening, and health screening results. The criteria for hypertension were I10–13 or I15 claim codes plus ≥ 1 prescription of an antihypertensive agent, or systolic/diastolic blood pressure ≥ 140/90 mmHg); the criteria for diabetes were E11–14 claim codes plus ≥ 1 prescription of an antidiabetic medication per year, or a fasting glucose level ≥ 126 mg/dL; and the criteria for dyslipidemia were E78 claim code plus ≥ 1 prescription of a lipid-lowering agent, or total cholesterol ≥ 240 mg/dL. Chronic kidney disease was defined as an estimated glomerular filtration rate < 60 mL/min/1.73 m^2^ (estimated using the Modification of Diet in Renal Disease equation).

Body mass index (BMI) was calculated as weight (kg) divided by the square of the person’s height (m). Waist circumference (WC) was measured by a well‐trained person at the midpoint between the lower costal margin and the iliac crest. After an overnight fast of ≥ 8 h, blood specimens were obtained from each participant.

### Surrogate measure of fatty liver

Although ultrasonography is a first-line screening technique in clinical practice^21^, ultrasonography is not included in the NHIS mass screening program. Therefore, biochemical data were used to identify NAFLD. These data were used to calculate the fatty liver index (FLI) score according to the below formula (in which GGT refers to gamma-glutamyl transferase)^22^. This score ranges from 0 to 100, with < 30 representing low risk of a fatty liver and ≥ 60 representing high risk of a fatty liver.

$${\text{FLI}}\, = \,{\text{e}}^{{0.{\text{953}} \times {\text{ln}}\,{\text{ triglyceride}} + 0.{\text{139}} \times {\text{BMI}} + 0.{\text{718}} \times {\text{ln }}\,{\text{GGT}} + 0.0{\text{53}} \times {\text{WC}}{-}{\text{15}}.{\text{745}}}} /\left( {{\text{1}}\, + \,{\text{e}}^{{0.{\text{953}} \times {\text{ln }}\,{\text{triglyceride}} + 0.{\text{139}} \times {\text{BMI}} + 0.{\text{718}} \times {\text{ln }}\,{\text{GGT}} + 0.0{\text{53}} \times {\text{WC}}{-}{\text{15}}.{\text{745}}}} } \right)\, \times \,{\text{1}}00.$$

### Study outcome

The study population was followed from baseline to the date of OSA diagnosis or until December 31, 2015, whichever occurred first. The primary endpoint of this study was incident OSA, which was defined as ≥ 1 one claim using the ICD‐10 code G473.

### Statistical analyses

Statistical analysis is similar to a previous study^23^. Data are presented as mean ± standard deviation for normally distributed continuous variables and as proportions for categorical variables, unless otherwise indicated. Student’s t-test and analysis of variance were used to analyze continuous variables, and differences between nominal variables were compared using the chi-square test. Log transformations were performed for non-normally distributed variables. Incidence rate of the primary outcome was calculated by dividing the number of incident cases by the total follow-up period and presented as per 1000 person-years. We constructed a Cox proportional hazard model (Model 1) adjusted for age and sex, and the multivariate analysis included more potential confounders: smoking, alcohol consumption, exercise^24^, income^25^, hypertension, dyslipidemia, diabetes and BMI (Model 2), WC instead of BMI (Model 3), and lipid accumulation product (LAP)^26^ instead of BMI or WC (Model 4). Statistical analyses were performed using SAS version 9.4 (SAS Institute, Cary, NC, USA) and R version 3.2.3 (The R Foundation for Statistical Computing, Vienna, Austria, http://www.Rproject.org). A two-sided P value < 0.05 was considered statistically significant.

## Results

### Baseline characteristics of the study population

The prevalence of NAFLD, based on an FLI ≥ 60, was 11.5%. The total population was divided into three groups according to FLI score: < 30, 30–60, and ≥ 60. The baseline characteristics of each group are shown in Table 1. Compared to the lowest FLI group, the higher FLI groups had higher rates of males, current smokers, alcohol consumers, and people with a higher income level. Hypertension, dyslipidemia, and diabetes mellitus were also associated with a higher FLI. Most anthropometric and laboratory variables (including BMI, WC, total cholesterol, triglycerides, high-density lipoprotein cholesterol, and fasting glucose) were less metabolically favorable in people in the higher FLI groups than in those in the lowest FLI group (P < 0.001).

**Table 1:** Baseline characteristics of participants according to fatty liver index.

	Fatty liver index			*P-*value
	0–30	30–60	≥ 60	
	N = 5,352,484	N = 1,833,489	N = 930,551	
Age, years, *n (%)*				< 0.0001
20–39	1,894,600 (35.4)	445,159 (24.3)	281,967 (30.3)	
40–64	2,825,766 (52.8)	1,082,556 (59.0)	541,620 (58.2)	
≥ 65	632,118 (11.8)	305,774 (16.7)	106,964 (11.5)	
Male, *n (%)*	2,187,100 (40.9)	1,270,740 (69.3)	756,910 (81.3)	< 0.0001
Smoking, *n (%)*				< 0.0001
Non-smoker	3,776,555 (70.6)	924,872 (50.4)	361,161 (38.8)	
Ex-smoker	552,462 (10.3)	340,835 (18.6)	185,316 (19.9)	
Current-smoker	1,023,467 (19.1)	567,782 (31.0)	384,074 (41.3)	
Alcohol consumption (yes), *n (%*)	2,184,391 (40.8)	937,241 (51.1)	580,070 (62.3)	< 0.0001
Regular exercise (yes),* n (%*)	931,252 (17.4)	343,617 (18.7)	159,076 (17.1)	< 0.0001
Income (lowest 20%), *n (%)*	1,533,373 (28.7)	441,547 (24.1)	217,947 (23.4)	< 0.0001
Waist circumference (cm)	75.52 ± 6.99	85.90 ± 5.38	92.12 ± 6.76	< 0.0001
Body mass index (kg/m^2^)	22.21 ± 2.41	25.52 ± 2.24	27.93 ± 2.99	< 0.0001
Systolic blood pressure (mmHg)	119.09 ± 14.32	126.52 ± 14.31	129.86 ± 14.49	< 0.0001
Diastolic blood pressure(mmHg)	74.13 ± 9.52	78.78 ± 9.52	81.44 ± 9.87	< 0.0001
Comorbidity, *n (%)*				
Diabetes	257,903 (4.8)	222,231 (12.1)	166,947 (17.9)	< 0.0001
Hypertension	927,512 (17.3)	647,256 (35.3)	927,512 (17.3)	< 0.0001
Dyslipidemia	654,800 (12.2)	466,174 (25.4)	316,916 (34.1)	< 0.0001
Chronic kidney disease	290,169 (5.4)	129,087 (7.0)	60,469 (6.5)	< 0.0001
Laboratory findings				
Serum glucose (mg/dL)	93.27 ± 18.00	100.74 ± 25.12	106.76 ± 31.44	< 0.0001
Total cholesterol (mg/dL)	189.14 ± 34.42	203.88 ± 36.62	212.52 ± 38.85	< 0.0001
HDL-cholesterol (mg/dL)	58.00 ± 17.61	51.27 ± 19.81	48.79 ± 19.99	< 0.0001
Triglyceride^a^ (mg/dL)	87.6 (87.57–87.63)	155.34 (155.25–155.44)	230.04 (229.81–230.27)	< 0.0001

Data are presented as mean ± standard deviation for continuous variables and n (%) for categorical variables.

*HDL* high density lipoprotein.

^a^Geometric means.

### Risk of incident OSA in people with NAFLD

During the mean 6.3 years of follow-up period, the OSA incidence rate was higher in subjects with a high (≥ 60) FLI than in those with a low (< 30) FLI (1.831 vs 0.567 outcomes per 1000 person-years, Table 2). After adjusting for age and sex, the risk of OSA was significantly higher in the higher FLI groups, compared with the lowest FLI group (hazard ratio [HR] 1.68, 95% confidence interval [CI] 1.64–1.72 for FLI 30–60 and HR 2.42, 95% CI 2.36–2.48 for FLI ≥ 60). After adjusting for age, sex, smoking, alcohol consumption, physical exercise, income, BMI, diabetes, hypertension, and dyslipidemia, these associations remained significant (adjusted hazard ratio [aHR] 1.15, 95% CI 1.12–1.18 for FLI 30–60 and aHR = 1.21, 95% CI 1.17–1.26 for FLI ≥ 60). When we adjusted waist circumference (Model 3), and LAP (Model 4) instead of BMI, an increased risk of OSA in the highest FLI group compared with the lowest FLI group, still observed (Table 2).

**Table 2:** Risk of obstructive sleep apnea according to fatty liver index.

Fatty liver index	No. of subjects	No. of events	Incidence rate (1000 person-years)	HR (95% CI)			
				Model 1	Model 2	Model 3	Model 4
0–30	5,352,484	19,134	0.56665	1 (reference)	1 (reference)	1 (reference)	1 (reference)
30–60	1,833,489	13,238	1.14744	1.68 (1.64, 1.72)	1.15 (1.12, 1.18)	1.23 (1.20, 1.27)	1.58 (1.54, 1.62)
60–	930,551	10,662	1.83065	2.42 (2.36, 2.48)	1.21 (1.17, 1.26)	1.38 (1.33, 1.42)	2.08 (2.05, 2.15)

Model 1: adjusted for age and sex.

Model 2: adjusted for Model 1 + smoking, alcohol consumption, exercise, income, hypertension, dyslipidemia, diabetes and body mass index.

Model 3: adjusted for Model 1 + smoking, alcohol consumption, exercise, income, hypertension, dyslipidemia, diabetes and waist circumference.

Model 4: adjusted for Model 1 + smoking, alcohol consumption, exercise, income, hypertension, dyslipidemia, diabetes and LAP.

*p-y* person-year, *HR* hazard ratio, *CI* confidence interval, *LAP* lipid accumulation product.

### OSA risk according to obesity, abdominal obesity, and other factors

When we stratified the population according to the BMI, the risk of OSA was significantly increased in higher FLI groups (FLI 30–60 and ≥ 60) in both the non-obese (BMI < 25), and the obese subjects (BMI ≥ 25) (Table 3). The risk of OSA was highest in patients with the highest FLI and obesity (aHR 2.58, 95% CI 2.51–2.66). When stratified according to WC, the risk of OSA was significantly increased in higher FLI groups (30–60 and ≥ 60) in patients with abdominal obesity (WC ≥ 90 cm for males and ≥ 80 cm for females), as well as in those without abdominal obesity. The risk of OSA was highest in patients with the highest FLI and abdominal obesity (aHR 2.60, 95% CI 2.53–2.68).

**Table 3:** Risk of obstructive sleep apnea according to fatty liver index according to obesity.

	FLI	No. of subjects	No. of events	Follow-up duration (person-year)	Incidence rate (per 1000 p-y)	HR (95% CI)
**Obesity**						
Absent	0–30	4,698,289	15,957	29,624,186	0.53865	1 (ref.)
	30–60	773,186	4825	4,850,755	0.99469	1.38 (1.34, 1.43)
	60-	134,163	864	835,797	1.03374	1.32 (1.23, 1.41)
Present	0–30	654,195	3177	4,142,761	0.76688	1.57 (1.51, 1.63)
	30–60	1,060,303	8413	6,686,262	1.25825	1.99 (1.93, 2.04)
	60-	796,388	9798	4,988,365	1.96417	2.58 (2.51, 2.66)
**Abdominal obesity**						
Absent	0–30	4,642,000	16,900	29,264,972	0.57748	1 (ref.)
	30–60	1,037,995	8429	6,515,871	1.29361	1.56 (1.51, 1.60)
	60-	283,220	2854	1,770,648	1.61184	1.80 (1.73, 1.88)
Present	0–30	710,484	2234	4,501,976	0.49623	1.51 (1.44, 1.58)
	30–60	795,494	4809	5,021,147	0.95775	1.92 (1.85, 1.98)
	60-	647,331	7808	4,053,513	1.92623	2.60 (2.53, 2.68)

Model: adjusted for age, sex, smoking, alcohol consumption, exercise, income, hypertension, dyslipidemia diabetes and body mass index.

*FLI* fatty liver index, *p-y* person-year, *HR* hazard ratio, *CI* confidence interval.

**Figure 1:**
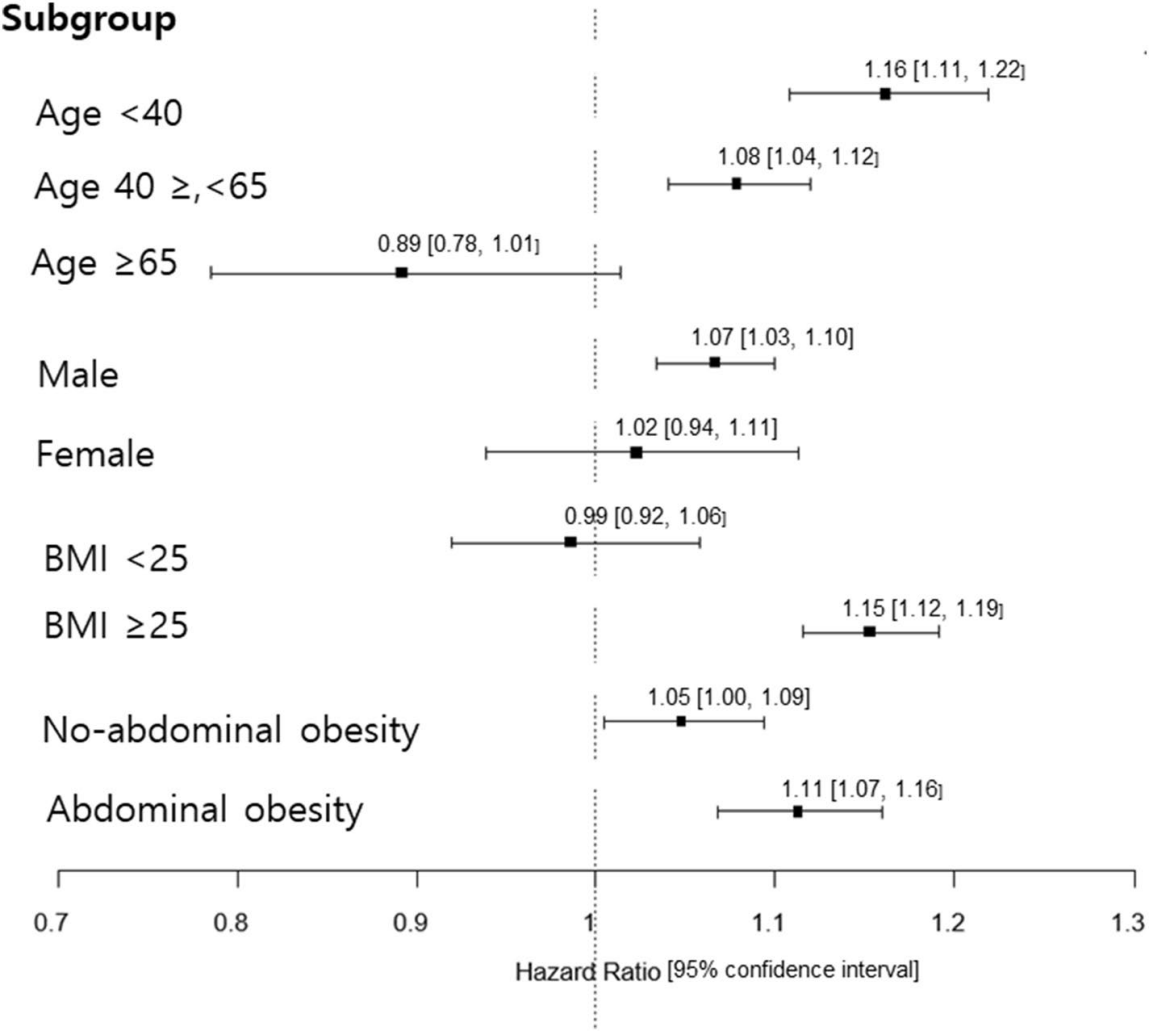
Forest plot shows the HRs of the association between FLI ≥ 60 and OSA in different subgroups of participants; age < 40, 40 ≤ age < 65 vs age ≥ 65 years, male vs female, nonobese (BMI < 25) vs obese (BMI ≥ 25), no-abdominal obesity vs abdominal obesity. HRs are represented by black square; 95% CI are denoted by horizontal whiskers. *FLI* fatty liver index, *OSA* obstructive sleep apnea, *BMI* body mass index, *HR* hazard ratio, *CI* confidence interval.

Next, we investigated various factors affecting the risk of OSA in patients with NAFLD (FLI ≥ 60). When stratified by age, sex, BMI, and abdominal obesity, the risk of OSA was significantly higher in NAFLD patients with younger age, male sex, obesity and abdominal obesity than in those with an older age, female sex, without obesity and abdominal obesity (Fig. 1).

## Discussion

In this nationwide population-based study involving most Korean adults, we found that NAFLD (defined by FLI) was significantly associated with increased risk of OSA, even after adjusting for multiple metabolic variables. Moreover, the risk of OSA was higher in NAFLD patients who were younger, male, or obese than in those who were older, female, or non-obese.

Many studies have shown that patients with OSA have a higher incidence of NAFLD than the normal population, suggesting a link between hepatic injury and hypoxia in OSA^15,17^. A French study of 1285 patients with OSA found a linear relationship between OSA severity and hepatic steatosis index^27^. The relationship was independent of confounding factors, thereby suggesting that liver damage may occur during OSA. Similarly, an in vitro study showed that chronic intermittent hypoxia in obese mice caused hyperlipidemia by inhibiting clearance of triglyceride-rich lipoproteins^28^.

To date, most studies have evaluated the increased risk of hepatic steatosis in patients with OSA. Few studies have examined the converse association: the risk of OSA in patients with hepatic steatosis. One study reported that the overall prevalence of OSA was significantly higher in patients with fibrosis, compared to those without fibrosis, in NAFLD patients with chronically elevated liver enzymes^29^. Similarly, the apnea–hypopnea index was higher in patients with hepatic fibrosis than in those without fibrosis in NAFLD patients with severe obesity^30^. Another study reported that polysomnographic parameters, including apnea–hypopnea index and oxygen desaturation index, were significantly higher in patients with moderate or severe NAFLD, compared with individuals without NAFLD^31^. A population-based study based on the United States National Health and Nutrition Examination Survey showed that NAFLD, defined by elevated liver enzymes, was associated with sleep disorders diagnosed using sleep disorder questionnaires^32^. In agreement with these previous results, we found that the risk of OSA increased in a dose-dependent manner as FLI increased, supporting the presence of a close link between OSA and NAFLD.

The major confounding variable when analyzing the link between NAFLD and OSA is obesity. Measures of obesity include BMI and WC, both of which are associated with an increased risk of OSA^33^. When we classified subjects according to BMI and WC, the association between FLI and OSA remained in both obese and non-obese subgroups and in both with and without abdominal obesity subgroups. Moreover, patients with the highest FLI and obesity had the highest risk of incident OSA, supporting the important role of obesity in the risk of OSA. Furthermore, the association between NAFLD (FLI ≥ 60) and OSA was significantly higher in patients with a younger age, male sex, or obesity than in patients with an older age, female sex, or no obesity. These results are consistent with those of a study performed at a tertiary center in India, which found that the prevalence of symptomatic OSA was predicted by male sex and obesity^18^.

Mechanisms underlying the association between NAFLD and OSA are not fully elucidated. Intermittent hypoxia during OSA reverses normal diurnal glucose variation and promotes pancreatic beta-cell replication, which may lead to insulin resistance^34^. Chronic intermittent hypoxia also upregulates the sympathetic nervous system and increases circulating free fatty acids, hepatic gluconeogenesis, and induces insulin resistance^35^. Conversely, obesity among patients with NAFLD may lead to upper airway collapse, leading to chronic intermittent hypoxia^36^. However, the exact mechanism for assigning causal relationship is still known.

Regarding clinical practice, this study provides new insights to suggest the risks NAFLD may have for the the development of sleep impaired breathing. Another strength of this study is that it is a large-scale nationwide cohort study. However, the study has some limitations. First, because of its population-based observational study design, the results should be interpreted cautiously. Second, using FLI as a surrogate marker of NAFLD cannot accurately quantify the presence and amount of steatosis^37^. Although liver biopsy is considered the gold standard for diagnosing NAFLD, it is not usually performed in healthy individuals. Radiographic techniques, such as ultrasonography or magnetic resonance imaging, are typically used for diagnosing NAFLD in clinical practice. However, FLI (≥ 60) was previously investigated as a surrogate marker for NAFLD and found to accurately diagnose steatosis and correlate with insulin resistance^37^. Third, because we could not obtain data from polysomnography, which is the gold standard diagnostic test for OSA^38^, we could not analyze the severity of OSA. Fourth, since the diagnosis of OSA was based on claims data using the ICD-10 code, the number of subjects with OSA may have been underestimated. Although OSA affects approximately 20% of US adults, of whom about 90% are undiagnosed. This may be due to poor awareness of OSA, a lack of routine screening, and the limited number of facilities for sleep study^39^. Lastly, the diagnostic method of sleep apnea may not be unified. However, the diagnosis of sleep apnea was usually done using polysomnography according to recently published data in Korea^40,41^. Since the aim of this study was not about the exact diagnosis of OSA, but to examine the relationship between clinically significant OSA and NAFLD, we used the ICD-code to define OSA as previous study^42^. Further investigations using more accurate measures to diagnose OSA are necessary to confirm our results.

In conclusion, a high FLI score may help identify individuals with a high risk of OSA. Further study should be done to identify the risk profile for OSA in NAFLD patients. Understanding the association between NAFLD and OSA may have clinical important implications for reducing the incidence of these comorbidities.

## Abbreviations

OSA: Obstructive sleep apnea
NAFLD: Nonalcoholic fatty liver disease
FLI: Fatty liver index
HR: Hazard ratio
CI: Confidence interval
NHIS: National Health Insurance System
ICD: International Classification of Diseases
BMI: Body mass index
WC: Waist circumference
GGT: Gamma-glutamyl transferase
LAP: Lipid accumulation product
